# Preparation and *in Vivo* Evaluation of a Dutasteride-Loaded Solid-Supersaturatable Self-Microemulsifying Drug Delivery System

**DOI:** 10.3390/ijms160510821

**Published:** 2015-05-13

**Authors:** Min-Soo Kim, Eun-Sol Ha, Gwang-Ho Choo, In-Hwan Baek

**Affiliations:** 1 College of Pharmacy, Pusan National University, Busan 609-735, Korea; E-Mails: edel@pusan.ac.kr (E.-S.H.); jimasd@pusan.ac.kr (G.-H.C.); 2 College of Pharmacy, Kyungsung University, Busan 608-736, Korea; E-Mail: baek@ks.ac.kr

**Keywords:** self-emulsifying, bioavailability, dutasteride, cellulose, drug delivery

## Abstract

The purpose of this study was to prepare a dutasteride-loaded solid-supersaturatable self-microemulsifying drug delivery system (SMEDDS) using hydrophilic additives with high oral bioavailability, and to determine if there was a correlation between the *in vitro* dissolution data and the *in vivo* pharmacokinetic parameters of this delivery system in rats. A dutasteride-loaded solid-supersaturatable SMEDDS was generated by adsorption of liquid SMEDDS onto Aerosil 200 colloidal silica using a spray drying process. The dissolution and oral absorption of dutasteride from solid SMEDDS significantly increased after the addition of hydroxypropylmethyl cellulose (HPMC) or Soluplus. Solid SMEDDS/Aerosil 200/Soluplus microparticles had higher oral bioavailability with 6.8- and 5.0-fold higher peak plasma concentration (C_max_) and area under the concentration-time curve (AUC) values, respectively, than that of the equivalent physical mixture. A linear correlation between *in vitro* dissolution efficiency and *in vivo* pharmacokinetic parameters was demonstrated for both AUC and C_max_ values. Therefore, the preparation of a solid-supersaturatable SMEDDS with HPMC or Soluplus could be a promising formulation strategy to develop novel solid dosage forms of dutasteride.

## 1. Introduction

**Figure 1:**
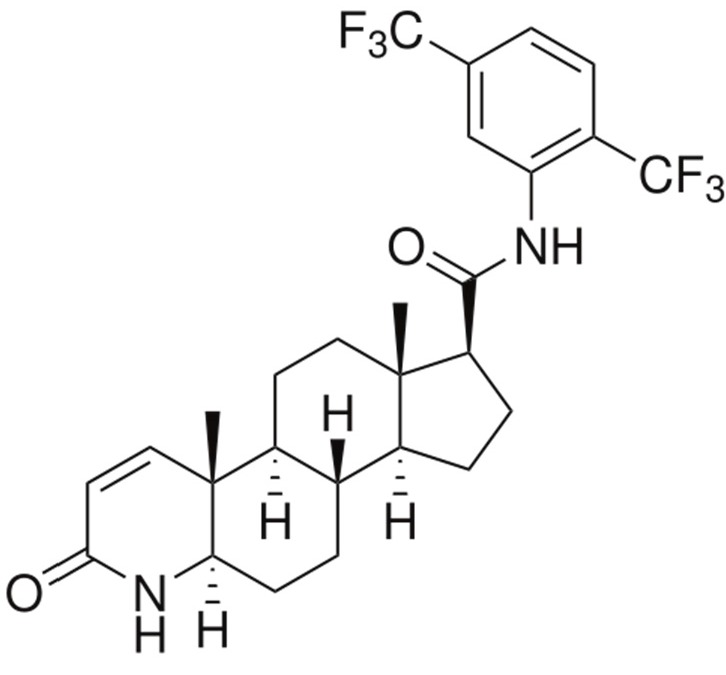
The chemical structure of dutasteride.

Dutasteride is a 5-α reductase inhibitor (Figure 1), which is used to treat benign prostatic hyperplasia [1]. In GlaxoSmithKline’s commercial formulation, Avodart^®^, dutasteride’s poor aqueous solubility (<0.038 ng/mL) is overcome by using mono-di-glycerides of caprylic/capric acid and butylated hydroxytoluene to form soft gelatin capsules containing 0.5 mg dutasteride. To develop a solid dutasteride dosage form with high oral bioavailability, we evaluated various formulations, such as Eudragit E nanosuspension, hydroxypropyl-β-cyclodextrin (HP-β-CD) nanostructures, silica nanomatrices, self-microemulsifying drug delivery systems (SMEDDS), and solid dispersions [2,3,4,5,6,7]. Among the solid formulations tested, the bioavailability of dutasteride-loaded HP-β-CD nanostructures with hydroxypropylmethyl cellulose (HPMC) prepared using a supercritical antisolvent (SAS) process was similar to that of the commercial product [7]. Unfortunately, it is difficult to scale up supercritical fluid technology in the pharmaceutical industry, because high-pressure equipment is required for product formulation.

SMEDDS, consisting of oil, surfactant and/or co-solvent, is being utilized to improve the oral bioavailability of API [8,9,10]. However, liquid SMEDDS formulations must be solidified using an adsorbent to improve stability, reproducibility, and patient compliance [11]. Spray drying is a one-step method employed to prepare solid forms of self-microemulsifying formulations using adsorbents, such as colloidal silica (Aerosil^®^ 200) and magnesium aluminometasilicate (Neusilin^®^) [12,13,14]. Unfortunately, the amount of dissolved drug gradually decreases owing to the decreased solubilizing capacity of SMEDDS after dilution in aqueous medium *in vitro* or in gastrointestinal fluid *in vivo*. Therefore, precipitation of the supersaturated dissolved drug induced by SMEDDS dispersion should be avoided. Supersaturatable SMEDDS can be obtained by using hydrophilic additives to inhibit precipitation [15,16,17,18]. Specifically, supersaturatable SMEDDS containing HPMC dramatically increased oral absorption of poorly water-soluble APIs, including paclitaxel, AMG 517, and PNU-91325, as compared to SMEDDS formulations without a precipitation inhibitor [19,20,21]. The selection of an appropriate hydrophilic additive needs to be based on experimental, approaches because reliable structure-activity relationships have not been established [22]. 

In our previous study, we developed and optimized a dutasteride-loaded SMEDDS formulation containing Capryol 90, Cremophor EL, and Transcutol HP, based on a d-optimal mixture design [5]. The purpose of this study was to develop a dutasteride-loaded solid-supersaturatable SMEDDS with high oral bioavailability, and to determine if there is a correlation between *in vitro* dissolution data and *in vivo* pharmacokinetic parameters in rats. Dutasteride-loaded solid-supersaturatable SMEDDS were prepared by adsorption of liquid SMEDDS onto Aerosil 200 colloidal silica using a spray drying process. The effect of various hydrophilic additives on the supersaturation, dissolution, and oral bioavailability of dutasteride were evaluated. *In vitro-in vivo* correlation (IVIVC) studies were also conducted.

## 2. Results and Discussion

**Figure 2:**
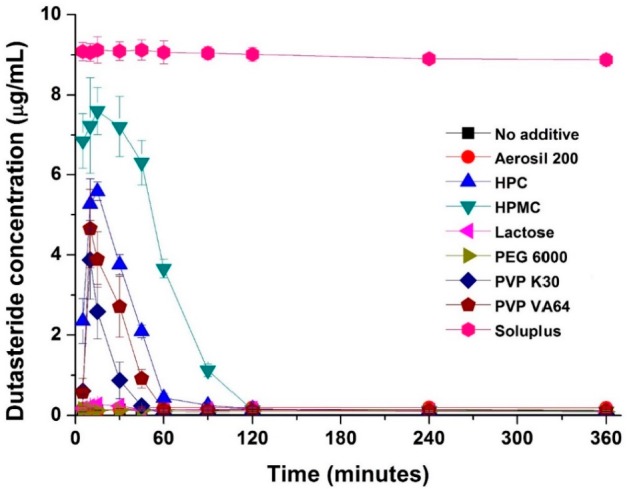
Effect of hydrophilic polymers on dutasteride recrystallization. Data are expressed as the mean ± standard deviation (*n* = 3).

In this study, dutasteride-loaded solid-supersaturatable SMEDDS was prepared by adsorption of liquid SMEDDS onto colloidal silica using a spray drying process. The effect of hydrophilic additives such as hydroxypropyl cellulose (HPC), HPMC, lactose, polyethylene glycol (PEG) 6000, polyvinylpyrrolidone (PVP) K30, PVP VA64, and Soluplus on the supersaturation, dissolution, and oral bioavailability of dutasteride was investigated. The inhibitory effect of hydrophilic additives on the recrystallization of dutasteride was investigated in pH 1.2 dissolution medium. As shown in Figure 2, dutasteride rapidly precipitated in pH 1.2 dissolution medium without hydrophilic additives. However, dutasteride recrystallization was significantly inhibited by hydrophilic additives. The most effective additive was Soluplus, followed by HPMC, HPC, PVP VA64, and finally PVP K30. Dutasteride concentration was maintained above 8 μg/mL for at least 6 h using Soluplus. Soluplus is a graft copolymer of polyvinyl caprolactam, polyvinyl acetate, and polyethylene glycol and is an effective solubilizing excipient for poorly soluble drugs since it allows formation of supersaturated polymeric micelles and a polymeric amorphous solid dispersion (Table S1) [23,24].

Dutasteride solubility was less than 1 μg/mL in pH 1.2 dissolution medium containing 1 mg/mL Soluplus. Therefore, the high concentration of dutasteride in solution cannot be entirely attributable to the solubilization properties of Soluplus. Rather, Soluplus, by blocking the active surface and providing steric hindrance, inhibits crystal nucleation and growth, which subsequently results in increased concentration dutasteride in solution [25,26].

Recently, it was reported that Soluplus can inhibit the recrystallization of poorly water-soluble APIs, such as atorvastatin and cyclosporine [24,26]. In this recrystallization study, Soluplus was found to be the most effective inhibitor of dutasteride recrystallization among the various hydrophilic additives tested.

**Figure 3:**
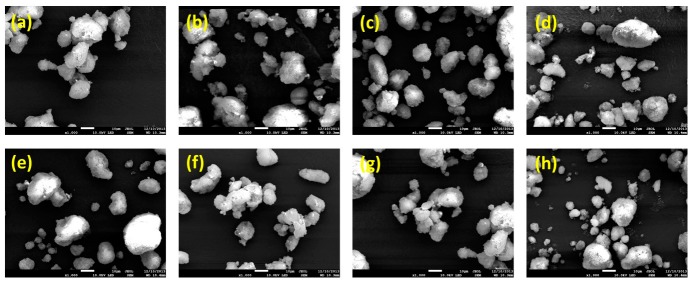
Scanning electron micrographs of dutasteride-loaded solid-supersaturatable self-microemulsifying drug delivery systems (SMEDDS): (**a**) SMEDDS:Aerosil 200; (**b**) SMEDDS:Aerosil 200:HPC; (**c**) SMEDDS:Aerosil 200:HPMC; (**d**) SMEDDS:Aerosil 200:lactose; (**e**) SMEDDS:Aerosil 200:PEG6000; (**f**) SMEDDS:Aerosil 200:PVP K30; (**g**) SMEDDS:Aerosil 200:PVA VA64; and (**h**) SMEDDS:Aerosil 200:Soluplus. Bar = 10 μm.

SEM observation (Figure 3) revealed that the spray drying process yielded well-fabricated solid SMEDDS microparticles. As shown in Table 1, all particles were irregularly shaped with similar volumes and mean particle sizes (7–9 μm). There was no significant size difference between solid SMEDDS microparticles (*p* > 0.05). These data indicate that the morphology of solid SMEDDS microparticles was not influenced by hydrophilic additives. The mean droplet size of the homogeneous microemulsion was 37.5 ± 3.7 nm following dilution of liquid SMEDDS in an aqueous solution. After dispersion of solid SMEDDS/Aerosil 200 microparticles in water, droplets with a mean diameter of 40.9 ± 5.5 nm were formed. There was no significant difference between liquid SMEDDS and solid SMEDDS/Aerosil 200. Aerosil 200 did not affect droplet formation from SMEDDS and was a good solid adsorbent for dutasteride-containing SMEDDS. The droplet size of SMEDDS was affected by the addition of hydrophilic additives to solid SMEDDS/Aerosil 200. Solid SMEDDS particles had a narrow droplet size distribution, with a mean droplet size of 43.9 ± 8.9 nm for SMEDDS/Aerosil 200/Soluplus and 50.3 ± 9.6 nm for SMEDDS/Aerosil 200/PVP VA64 powder. SMEDDS/Aerosil 200/HPC and SMEDDS/Aerosil 200/PEG 6000 particles had a broader size distribution, with a mean droplet size of 133.7 ± 22.6 and 175.7 ± 24.6 nm, respectively. Among the various hydrophilic additives tested, Solpulus was considered the best additive for generating the smallest droplet size from SMEDDS/Aerosil 200.

**Table 1:** Formulation and particle size of dutasteride-loaded solid-supersaturatable SMEDDS.

Formulation	Volume Mean Particle Size ^a^ (μm)	Mean Droplet Size ^b^ (nm)
Liquid SMEDDS	ND	37.5 ± 3.7 (0.141) ^d^
SMEDDS:Aerosil 200 = 1:1	7.63 ± 2.01 (1.98) ^c^	40.9 ± 5.5 (0.189)
SMEDDS:Aerosil 200:HPC = 1:1:1	8.19 ± 1.93 (1.91)	133.7 ± 22.6 (0.243)
SMEDDS:Aerosil 200:HPMC = 1:1:1	7.52 ± 2.11 (2.11)	94.2 ± 10.5 (0.221)
SMEDDS:Aerosil 200:lactose = 1:1:1	8.01 ± 1.22 (1.89)	55.5 ± 7.7 (0.198)
SMEDDS:Aerosil 200:PEG6000 = 1:1:1	9.02 ± 2.22 (2.12)	175.7 ± 24.6 (0.246)
SMEDDS:Aerosil 200:PVP K30 = 1:1:1	8.44 ± 1.56 (1.89)	69.3 ± 8.5 (0.179)
SMEDDS:Aerosil 200:PVA VA64 = 1:1:1	8.78 ± 1.88 (1.97)	50.3 ± 9.6 (0.206)
SMEDDS:Aerosil 200:Soluplus = 1:1:1	7.49 ± 1.72 (1.95)	43.9 ± 8.9 (0.195)

^a^ Particle size of the dutasteride-loaded solid-supersaturatable SMEDDS powder in the solid state was measured using a HELOS laser diffraction analyzer; ^b^ Droplet size of the dutasteride-loaded solid-supersaturatable SMEDDS in the redispersed state was measured using dynamic light scattering techniques; ^c^ SPAN = (*d*_90_−*d*_10_)/*d*_50_, where *d*_10_, *d*_50_, and *d*_90_ are the diameter sizes and the given percentage value is the percentage of the particles smaller than that size; ^d^ The polydispersity index as an estimation of the particle size distribution width is dimensionless and is scaled between 0 and 1. Data are expressed as the mean ± standard deviation (*n* = 3).

**Figure 4:**
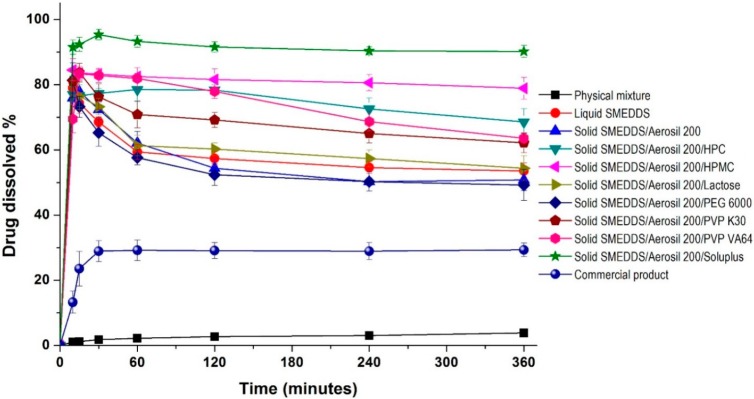
Dissolution profiles of dutasteride-loaded solid-supersaturatable SMEDDS in pH 1.2 dissolution medium. Data are expressed as the mean ± standard deviation (*n* = 3).

The effect of hydrophilic additives on the dissolution of dutasteride from solid SMEDDS was evaluated in pH 1.2 dissolution medium. As shown in Figure 4, the maximum release of dutasteride from liquid SMEDDS was 79.0% within 0.5 h, and gradually decreased to 53.5% at 2 h. The dissolution profile of solid SMEDDS/Aerosil 200 microparticles was similar to that of liquid SMEDDS. The dissolution of dutasteride significantly increased upon the addition of hydrophilic additives to the solid SMEDDS/Aerosil 200 microparticles. For solid SMEDDS/Aerosil 200/HPMC microparticles, the dissolution of dutasteride was not altered for at least 6 h, with a maximum dissolution of 84.5%. Interestingly, complete dissolution of dutasteride was observed in solid SMEDDS/Aerosil 200/Soluplus microparticles, where dutasteride dissolution exceeded 90% at 6 h. However, none of the other hydrophilic additives tested exceeded a dissolution rate of 65% at 6 h. Analysis of variance (ANOVA) showed significant differences among the hydrophilic additives (*p* < 0.05), which were ranked by the Student-Newman-Keuls (SNK) test in the order of increasing dissolution percentages of dutasteride at 6 h as follows: Lactose = PEG 6000 < PVP K30 = PVP VA64 = HPC < HPMC < Soluplus (hydrophilic additives). The enhanced dissolution of dutasteride from SMEDDS was attributed to the inhibition of dutasteride recrystallization by hydrophilic additives, such as HPMC and Soluplus.

**Figure 5:**
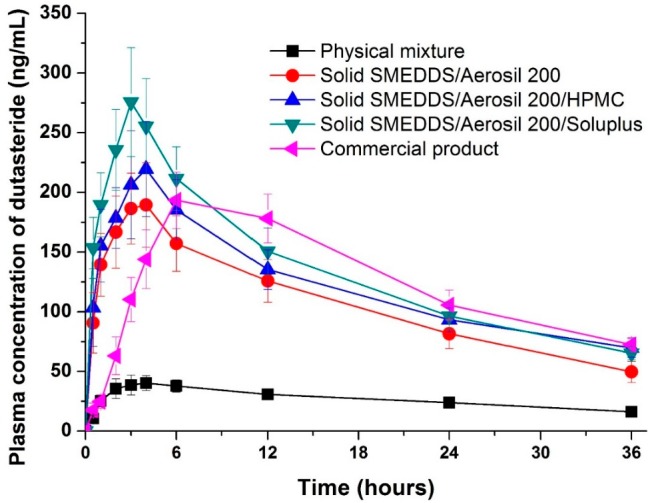
Plasma concentration-time profile for dutasteride in rats after oral administration of the physical mixture, dutasteride-loaded solid-supersaturatable SMEDDS, or commercial product. Data are expressed as the mean ± standard deviation (*n* = 5).

The pharmacokinetics of the physical mixture (raw material), dutasteride-loaded solid supersaturatable SMEDDS, and commercial product were examined in Sprague Dawley (SD) rats. The oral bioavailability of dutasteride from the solid-supersaturatable SMEDDS was much higher than from the physical mixture (Figure 5 and Table 2). Solid SMEDDS/Aerosil 200/Soluplus microparticles had a higher oral bioavailability than the physical mixture, displaying a 6.8- and 5.0-fold increase in maximum plasma concentration (C_max_) and area under the concentration-time curve (AUC_0→36 h_) values, respectively. The C_max_ of dutasteride increased in the following order: physical mixture < SMEDDS/Aerosil 200 = commercial product < SMEDDS/Aerosil 200/HPMC < SMEDDS/Aerosil 200/Soluplus. The solid SMEDDS/Aerosil 200/Soluplus microparticles had a higher mean AUC value than that of the commercial product; however, the difference was not statistically significant. The AUC value of solid SMEDDS/Aerosil 200/Soluplus microparticles was also similar to that of the commercial product. However, the AUC value of the solid SMEDDS/Aerosil 200 microparticles was significantly lower than that of the commercial product. These data show that the development of new solid dutasteride dosage forms can be achieved by solid-supersaturatable SMEDDS using HPMC or Soluplus.

**Table 2:** Dissolution efficiency and pharmacokinetic parameters for physical mixture, dutasteride-loaded solid-supersaturatable SMEDDS, and commercial product.

Formulation	DE ^a^ (%)	AUC_0→36 h_^b^ (ng·h/mL)	C_max_^b^ (ng/mL)	T_max_^b^ (h)
Physical mixture	2.8 ± 0.1	970.2 ± 122.5	40.2 ± 7.4	5.2 ± 1.1
SMEDDS:Aerosil 200 = 1:1	54.8 ± 1.9	3817.6 ± 399.9 ^c^	193.3 ± 15.0 ^c^	4.2 ± 1.1
SMEDDS:Aerosil 200:HPMC = 1:1:1	80.1 ± 2.1	4379.3 ± 420.8 ^cd^	222.7 ± 18.1 ^c−e^	4.0 ± 1.2
SMEDDS:Aerosil 200:Soluplus = 1:1:1	90.1 ± 1.1	4832.5 ± 540.5 ^cd^	275.2 ± 26.9 ^c−f^	3.0 ± 0.7
Commercial product	ND	4488.9 ± 490.4 ^cd^	193.2 ± 10.9 ^c^	6.8 ± 3.0

^a^ Data are expressed as the mean ± standard deviation (*n* = 3); ^b^ Data are expressed as the mean ± standard deviation (*n* = 5); ^c^ indicates *p* < 0.05 *vs.* physical mixture; ^d^ indicates *p* < 0.05 *vs.* SMEDDS:Aerosil 200; ^e^ indicates *p* < 0.05 *vs.* Commercial product; ^f^ indicates *p* < 0.05 *vs.* SMEDDS:Aerosil 200:HPMC.

**Figure 6:**
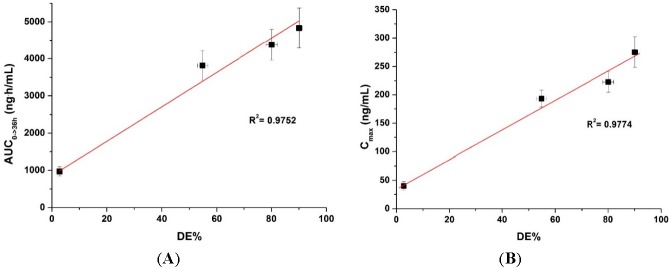
Figure 6. Correlation between the *in vitro* dissolution efficiency and *in vivo* pharmacokinetic parameters. (**A**) AUC and (**B**) C_max_.

**Figure 7:**
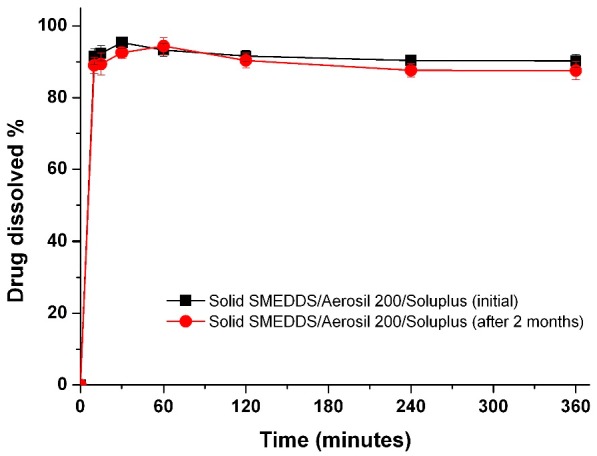
Dissolution profiles of the solid SMEDDS/Aerosil 200/Soluplus at pH 1.2 after 2 months under long-term conditions. Data are expressed as the mean ± standard deviation (*n* = 3).

To determine if there is a correlation between the *in vitro* dissolution data and *in vivo* pharmacokinetic parameters, the dissolution efficiency (DE%), as defined by Khan and Rhodes, was calculated (Table 2) [27]. As shown in Figure 6, a linear correlation between the *in vitro* dissolution efficiency and *in vivo* pharmacokinetic parameters was detected for both AUC and C_max_ values (*R*^2^ > 0.90). To improve the oral bioavailability of dutasteride, it is important that a high concentration of dutasteride is maintained for a long period of time *in vitro.* These results were similar to our previous IVIVC study using dutasteride-loaded HP-β-CD nanostructures prepared using the SAS process [7]. IVIVC studies using DE are recommended for poorly water-soluble APIs as previously reported [28,29,30]. Furthermore, the dutasteride content within solid SMEDDS/Aerosil 200/Soluplus was 97.2% ± 3.1% after two months at 25 ± 2 °C and 60% ± 3% relative humidity, and there were no significant changes compared to the drug content of the initial sample (101.3% ± 2.8%, *p* > 0.05). As shown in Figure 7, the dissolution profiles of dutasteride-loaded solid SMEDDS/Aerosil 200/Soluplus were similar to the drug content of the initial sample and the two-month-old samples. Therefore, the dutasteride-loaded solid SMEDDS/Aerosil 200/Soluplus is stable for at least two months at 25 ± 2 °C and 60% ± 3% relative humidity, which was considered long-term storage.

Generally, SMEDDS, consisting of oil, surfactant, and/or co-solvent (co-surfactant), provide a good solvent for poorly water-soluble APIs, with ultra-low interface tensions and large oil/water interface areas, allowing for API incorporation into oil droplets. When SMEDDS are placed in aqueous dissolution medium *in vitro* or gastrointestinal fluid *in vivo*, homogeneous oil-in-water microemulsions are spontaneously formed by mild agitation. Increased dissolution of APIs was observed initially, followed by a gradual decrease in the dissolved drug amount, owing to the reduction in solubilizing capacity due to dilution in *in vitro* aqueous dissolution medium or *in vivo* gastrointestinal fluid. Therefore, precipitation should be prevented for oral API administration. In many cases, the precipitation of poorly water soluble APIs in a supersaturated state can be controlled by hydrophilic additives. Specifically, HPMC may inhibit drug nucleation and crystal growth by blocking the active surface and providing steric stabilization in a supersaturated state, resulting in the dispersion of SMEDDS, which enhances oral absorption of poorly water-soluble APIs, such as paclitaxel, AMG 517, and PNU-91325 [19,20,21]. Soluplus, an amphiphilic polymer consisting of hydrophilic polyethylene glycol/polyvinyl acetate and hydrophobic polycaprolactam may also inhibit drug nucleation and crystal growth as described above. It was reported previously that amphiphilic polymers, such as pluronic acid, could sterically stabilize micellar structures and colloidal systems by acting like a surfactant [31,32,33]. The inhibition of precipitation may also be owing to specific interactions between dutasteride and HPMC or Soluplus, such as hydrophobic interactions or hydrogen bonding [34,35]; however, this requires further study. Taken together, the prolonged supersaturated state of dutasteride induced by HPMC or Soluplus resulted in increased oral absorption through the gastrointestinal epithelial membrane with an SMEDDS formulation. Furthermore, the solid-supersaturatable SMEDDS formulation using HPMC or Soluplus has utility for the formulation of APIs with poor aqueous solubility.

## 3. Experimental Section

### 3.1. Materials

Dutasteride was obtained from Reddy’s Laboratories Ltd. (Andhra Pradesh, India), finasteride from Sigma-Aldrich (St. Louis, MO, USA), and Aerosil 200 from Evonik Degussa (Hanau, Germany). Cremophor EL (polyoxyl 35 hydrogenated castor oil), polyvinylpyrrolidone (PVP K30), polyvinylpyrrolidone vinyl acetate (PVP-VA 64), and Soluplus (polyvinyl caprolactam-polyvinyl acetate-polyethylene glycol graft copolymer) were kindly supplied by BASF Co., Ltd. (Ludwigshafen, Germany). Capryol 90 (propylene glycol monocaprylate) and Transcutol HP (highly purified diethylene glycol monoethyl ether) were kindly donated by Gattefosse (Lyon, France). Hydroxypropylmethyl cellulose (HPMC 2910) and hydroxypropyl cellulose (HPC) were supplied by Shin-Etsu Chemical Co., Ltd. (Tokyo, Japan). Avodart^®^ soft gelatin capsule, which is a commercial 0.5 mg dutasteride product containing mono-di-glycerides of caprylic/capric acid and butylated hydroxytoluene for solubilization was purchased from GlaxoSmithKline (Brentford, UK). Ethanol, methanol, and acetonitrile were of HPLC grade.

### 3.2. Effect of Hydrophilic Additives on Dutasteride Recrystallization in a Supersaturated Solution

To investigate the effect of hydrophilic additives on recrystallization of a supersaturated dutasteride solution, dutasteride was dissolved in methanol (5 mg/mL). Dutasteride solution (1 mL) was added to of pH 1.2 dissolution medium (500 mL; HCl and NaCl) containing various hydrophilic additives (0.1% *w*/*w*), and agitated with a USP rotating paddle apparatus (Electrolab, Mumbai, India) at 37 ± 0.1 °C and 150 rpm. Samples (2 mL) were removed at various time intervals and filtered using a 0.22 μm syringe filter (nylon). The filtered samples were diluted with methanol, and the concentration of dutasteride was determined by HPLC analysis using a Waters HPLC system (Milford, MA, USA). Sample (20 μL) was injected into a C18 analytical column (Luna C18 [2]; 5 μm, 4.6 mm × 250 mm; Phenomenex, Torrance, CA, USA) using an autosampler. Separation and elution were achieved with a 60:40 mixture of acetonitrile and water, with an eluent flow rate of 1.0 mL/min. Dutasteride was detected by measuring the UV absorbance at a wavelength of 210 nm.

### 3.3. Preparation of Drug-Loaded Solid-Supersaturatable SMEDDS

Dutasteride-loaded liquid SMEDDS were prepared with Capryol 90, Cremophor EL, and Transcutol HP (35:30:35, *w*/*w*/*w*) based on the method described in our previous study [5]. Dutasteride (100 mg) was dispersed in the above-described mixture (20.0 g), and the components were mixed with gentle stirring until a clear solution was obtained. For preparation of dutasteride-loaded solid-supersaturatable SMEDDS, Aerosil 200 (2 g) was suspended in ethanol (400 mL), with or without hydrophilic additives (2 g), including PVP K30, PVP VA64, PEG6000, and Soluplus, by sonication for 30 min. For HPMC, HPC, and lactose, Aerosil 200 was dispersed in water:ethanol (3:7, *v*/*v*). The liquid SMEDDS (2.01 g) containing 10 mg dutasteride was added with constant stirring until a stable suspension was obtained. This suspension was spray dried using a mini spray dryer (B-191; Buchi, Flawil, Switzerland) under the following conditions: inlet temperature, 75–105 °C; outlet temperature, 55–75 °C; feed rate, 2–5 mL/min; and atomization air pressure, 5 kPa. The final drug content of the solid-supersaturatable SMEDDS formulation was 0.17% *w*/*w*. For the dissolution study using the raw material (dutasteride), the physical mixture consisted of raw material, Aerosil 200, and Soluplus (1:1:1, *w*/*w*/*w*) which was prepared by simple mixing using spatula in glass vial. The raw material did not dissolve in the dissolution medium without surfactant, due to its very low solubility.

### 3.4. Characterization of Drug-Loaded Solid-Supersaturatable SMEDDS

The morphology of solid-supersaturatable SMEDDS particles was observed using SEM (JSM-7100f; Jeol Ltd., Tokyo, Japan). The particle size and distribution were characterized using an HELOS laser diffraction spectrometer (SYMPATEC Ltd., Clausthal-Zellerfeld, Germany). For droplet size measurements, liquid SMEDDS or solid SMEDDS (equivalent to 0.5 mg of dutasteride) were dispersed by gentle mixing (10 s) in distilled water (25 mL). The resulting emulsion was incubated for 30 min at room temperature before samples were withdrawn for droplet size measurement. The droplet size was measured using dynamic light scattering (DLS; BI-9000; Brookhaven, Holtsville, NY, USA). The dissolution profiles of dutasteride from various dosage forms containing 0.5 mg dutasteride were determined using a USP rotating paddle apparatus (Electrolab, Mumbai, India) at 37 °C and 50 rpm in 500 mL of dissolution medium (HCl and NaCl; pH 1.2) [36]. Samples (3 mL) were collected at pre-determined intervals for analysis and replaced with fresh dissolution medium (3 mL) after each sample collection. Samples were filtered using a 0.05 μm syringe filter followed by dilution with methanol. The amount of drug dissolved in each sample was determined by HPLC.

### 3.5. Pharmacokinetic Study in Rats

Male Sprague-Dawley (SD) rats (250 ± 10 g; Orient Bio Inc., Seongnam, Korea) were divided into five treatment groups of five rats each. Prior to the study, the rats were fasted for 18 h and each group received physical mixture, solid supersaturatable SMEDDS, or commercial product at dutasteride doses of 2 mg/4 mL/kg (dose/water/rat weight) for each formulation by oral administration. SMEDDS:Aerosil 200, SMEDDS:Aerosil 200:HPMC, and SMEDDS:Aerosil 200:Soluplus formulation were selected based on the results of the recrystallization and dissolution studies. Physical mixture and solid supersaturatable SMEDDS were dispersed in water (1 mL) immediately prior to oral dosing. For the commercial product, the rats received the liquid content equivalent of 0.5 mg dutasteride followed immediately by administration of 1 mL of water. Blood samples (~0.35 mL) were collected in heparinized tubes from the retro-orbital plexus of rats at 0, 0.5, 1, 2, 3, 4, 6, 12, 24, and 36 h after dosing. Plasma samples were obtained by centrifugation at 10,000 rpm for 5 min (4 °C). Dutasteride plasma concentrations were determined by liquid chromatography with tandem mass spectrometry (LC-MS/MS), according to our previously reported method [2]. Pharmacokinetic analysis of the data was carried out with WinNonlin Standard Edition software, version 5.3 (Pharsight Corp., St. Louis, MO, USA). The area under the curve (AUC_0__→__36 h_) was calculated according to the trapezoidal method. The study protocol was in compliance with the institutional guidelines for the care and use of laboratory animals, and it was approved by the ethics committee of Kyungsung University (Busan, Korea).

### 3.6. Statistical Analysis

Statistical analysis for dissolution data and pharmacokinetic parameters was performed using a one-way analysis of variance (ANOVA) test followed by the Student-Newman-Keuls (SNK) and least-squares difference (LSD) tests with SPSS 21.0 software (IBM SPSS Statistics, Armonk, NY, USA).

## 4. Conclusions

In this study, dutasteride-loaded solid-supersaturatable SMEDDS was generated by the adsorption of liquid SMEDDS onto Aerosil 200 colloidal silica using a spray drying process. The dissolution and oral absorption of dutasteride from solid SMEDDS was increased by addition of HPMC or Soluplus. In conclusion, the preparation of solid-supersaturatable SMEDDS with HPMC or Soluplus using a spray drying process could be a promising formulation strategy for the development of new solid dosage forms of dutasteride.

## Supplementary Materials

Supplementary materials can be found at http://www.mdpi.com/1422-0067/16/05/10821/s1.
