# Supplementary material for: Preparation and in Vivo Evaluation of a Dutasteride-Loaded Solid-Supersaturatable Self-Microemulsifying Drug Delivery System
Source: Int J Mol Sci. 2015 May 13;16(5):10821–33. doi: 10.3390/ijms160510821 (PMC4463677; doi:10.3390/ijms160510821)
Supplement: Supplementary file 1 [file ijms-16-10821-s001.pdf]

Supplementary Information

Table S1. The chemical name, molecular structure and classification of excipient used.

Excipient	Chemical Name	Molecular Structure	Classification
Soluplus	polyvinyl caprolactam–polyvinyl acetate–polyethylene glycol graft copolymer	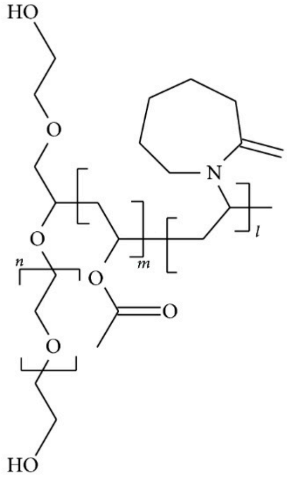	Polymeric solubilizer
Cremophor EL	polyoxyl 35 hydrogenated castor oil	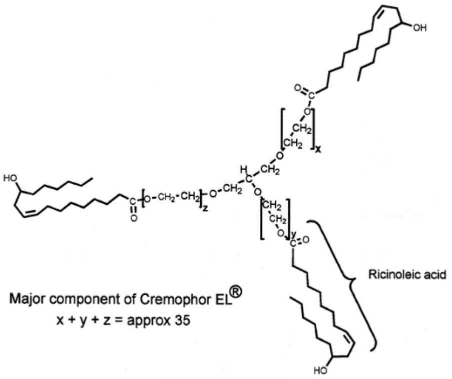	Surfactant
PVP K30	polyvinylpyrrolidone	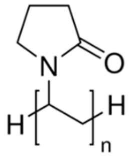	Hydrophilic polymer
PVP-VA 64	polyvinylpyrrolidone vinyl acetate	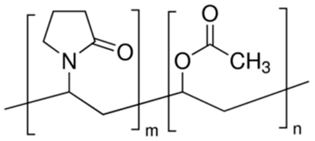	Hydrophilic polymer
Capryol 90	propylene glycol monocaprylate	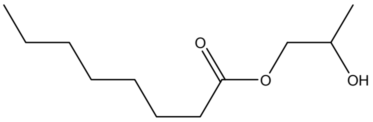	Oil
Transcutol HP	highly purified diethylene glycol monoethyl ether	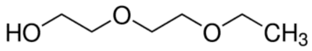	Surfactant

Table S1. *Cont.*

Excipient	Chemical Name	Molecular Structure	Classification
HPMC 2910	hydroxypropyl methylcellulose	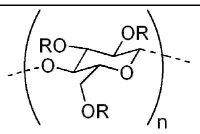 R = H or CH_3 or $CH_2CH(OH)CH_3$	Hydrophilic polymer
HPC	hydroxypropyl cellulose	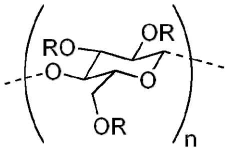 R = H or $CH_2CH(OH)CH_3$	Hydrophilic polymer
PEG 6000	polyethylene glycol 6000	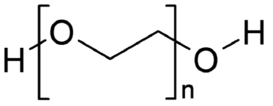	Hydrophilic polymer
Lactose	β-D-galactopyranosyl-(1→4)-D-glucose	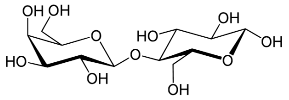	Diluent
